# A Mouse Model of Familial ALS Has Increased CNS Levels of Endogenous Ubiquinol_9/10_ and Does Not Benefit from Exogenous Administration of Ubiquinol_10_

**DOI:** 10.1371/journal.pone.0069540

**Published:** 2013-07-23

**Authors:** Jacopo Lucchetti, Marianna Marino, Simonetta Papa, Massimo Tortarolo, Giovanna Guiso, Silvia Pozzi, Valentina Bonetto, Silvio Caccia, Ettore Beghi, Caterina Bendotti, Marco Gobbi

**Affiliations:** 1 Department of Biochemistry and Molecular Pharmacology, IRCCS - Istituto di Ricerche Farmacologiche Mario Negri, Milano, Italy; 2 Department of Neuroscience, IRCCS - Istituto di Ricerche Farmacologiche Mario Negri, Milano, Italy; 3 Dulbecco Telethon Institute, IRCCS - Istituto di Ricerche Farmacologiche Mario Negri, Milano, Italy; University of Florida, United States of America

## Abstract

Oxidative stress and mitochondrial impairment are the main pathogenic mechanisms of Amyotrophic Lateral Sclerosis (ALS), a severe neurodegenerative disease still lacking of effective therapy. Recently, the coenzyme-Q (CoQ) complex, a key component of mitochondrial function and redox-state modulator, has raised interest for ALS treatment. However, while the oxidized form ubiquinone_10_ was ineffective in ALS patients and modestly effective in mouse models of ALS, no evidence was reported on the effect of the reduced form ubiquinol_10_, which has better bioavailability and antioxidant properties. In this study we compared the effects of ubiquinone_10_ and a new stabilized formulation of ubiquinol_10_ on the disease course of SOD1^G93A^ transgenic mice, an experimental model of fALS. Chronic treatments (800 mg/kg/day orally) started from the onset of disease until death, to mimic the clinical trials that only include patients with definite ALS symptoms. Although the plasma levels of CoQ_10_ were significantly increased by both treatments (from <0.20 to 3.0–3.4 µg/mL), no effect was found on the disease progression and survival of SOD1^G93A^ mice. The levels of CoQ_10_ in the brain and spinal cord of ubiquinone_10_- or ubiquinol_10_-treated mice were only slightly higher (≤10%) than the endogenous levels in vehicle-treated mice, indicating poor CNS availability after oral dosing and possibly explaining the lack of pharmacological effects. To further examine this issue, we measured the oxidized and reduced forms of CoQ_9/10_ in the plasma, brain and spinal cord of symptomatic SOD1^G93A^ mice, in comparison with age-matched SOD1^WT^. Levels of ubiquinol_9/10_, but not ubiquinone_9/10_, were significantly higher in the CNS, but not in plasma, of SOD1^G93A^ mice, suggesting that CoQ redox system might participate in the mechanisms trying to counteract the pathology progression. Therefore, the very low increases of CoQ_10_ induced by oral treatments in CNS might be not sufficient to provide significant neuroprotection in SOD1^G93A^ mice.

## Introduction

Coenzyme Q (CoQ) is an amphipathic molecule structurally composed of a quinone ring synthesized from *p*-hydroxybenzoate and of a polyisoprene chain synthesized from acetil-CoA [1]. There are different forms of CoQ, related to the length of polyisoprene chain: the predominant form in humans is CoQ_10_ which contains 10 isoprenoid units, whereas CoQ_9_ is the predominant form in rodents [2]. CoQ exists in three redox states: a fully oxidized form (ubiquinone), a fully reduced form (ubiquinol) and an intermediate semiquinone radical (CoQ^.−^). The redox state is tissue-specific: for example, in mice ubiquinol is the predominant form in liver and skeletal muscle while ubiquinone is predominant in the brain [3]. Several factors, such as drugs, age, diet and pathologies affect total levels and redox state of CoQ [4], [5], [6], [7] and their alterations may be regarded as markers of oxidative stress and mitochondrial dysfunctions [8].

Amyotrophic Lateral Sclerosis (ALS) is a severe neurodegenerative disorder in which the loss of motor neurons induces a progressive motor paralysis associated with dysphagia, dysarthria, respiratory failure and death within 3–5 years from the diagnosis. Analysis of plasma [9] and cerebrospinal fluid (CSF) [10] of sporadic ALS (sALS) patients have shown an increase of ubiquinone_10_ with a shift in the redox state of the coenzyme, interpreted as an index of oxidative stress.

Because of the substantial involvement of oxidative stress and mitochondrial impairment in this disease [11] different therapeutic approaches have been aimed towards counteracting these mechanisms. Among them, the use of CoQ_10_ has raised some interest for the treatment of ALS. In fact, on the basis of a modest but significant increase of survival observed in a mouse model of familial ALS (fALS) after chronic treatment with ubiquinone_10_ [12], a phase II trial was designed to investigate the effects of a chronic treatment with the same molecule (2700 mg daily) in patients with sALS. Unfortunately, the data collected after 9 months of treatment did not provide sufficient evidence of a benefit to justify a phase III trial [13].

The use of the reduced form ubiquinol, characterized by potent antioxidant properties, was difficult because it is readily oxidized in air. Recently, however, a new stabilized formulation of ubiquinol_10_ has been produced (Kaneka QH™) which overcomes this problem [14]. Notably, pharmacokinetic studies showed that ubiquinol_10_ has a better oral bioavailability than ubiquinone_10_, in humans [14], dogs and rats [15]. It may be expected that chronic treatment with this formulation results in higher CNS levels of CoQ_10_, with superior neuroprotective properties. Consistently, ubiquinol_10_ was more active than ubiquinone_10_ in protecting mice from striatal neurodegeneration induced by acute treatment with 1-methyl-4-phenyl-1,2,3,6-tetrahydropyridine (MPTP), a model for Parkinson’s disease [16].

On this basis, in the present study we evaluated: 1) the effects of a chronic oral administration with ubiquinol_10_ or ubiquinone_10_ on disease progression of transgenic SOD1^G93A^ mice, a model of fALS [11], 2) the endogenous levels of total, reduced and oxidized CoQ_10_ and CoQ_9_ in plasma, brain and lumbar spinal cord of transgenic SOD1^G93A^ mice at symptomatic stage.

Our data show that exogenous administration of ubiquinol_10_ or ubiquinone_10_ to SOD1^G93A^ mice, starting at the onset of symptoms, had no effect on the disease course. Since the basal levels of reduced CoQ_9/10_ are increased in the CNS of symptomatic SOD1^G93A^ mice, suggesting an attempt of equilibrium toward a protective antioxidant state, we think that the small increase of ubiquinol after the oral treatment is insufficient to exacerbate this effect.

## Materials and Methods

### Animals and Treatments

Transgenic SOD1^G93A^ and SOD1^WT^ mice on 129S2/SvHsd genetic background, and non-transgenic littermates were used in this study. The SOD1^G93A^ mouse line derives from the line originally obtained from Jackson Laboratories (B6SJL-TgNSOD-1-SOD1G93A-1Gur) expressing approximately 20 copies of mutant human SOD1 with a Gly93Ala substitution (SOD1^G93A^) or wild-type human SOD1 (SOD1^WT^) and were maintained on 129S2/SvHsd background for more than 15 generations at Harlan Italy S.R.L., Bresso (MI), Italy [17]. We observed no differences between the males and females as regards the disease progression and survival, as reported in supporting figure 1 (Fig. S1). Therefore, to reduce at minimum the number of transgenic mice used, chronic treatments were carried out in female whereas the biochemical profile was examined in males.

**Figure 1:**
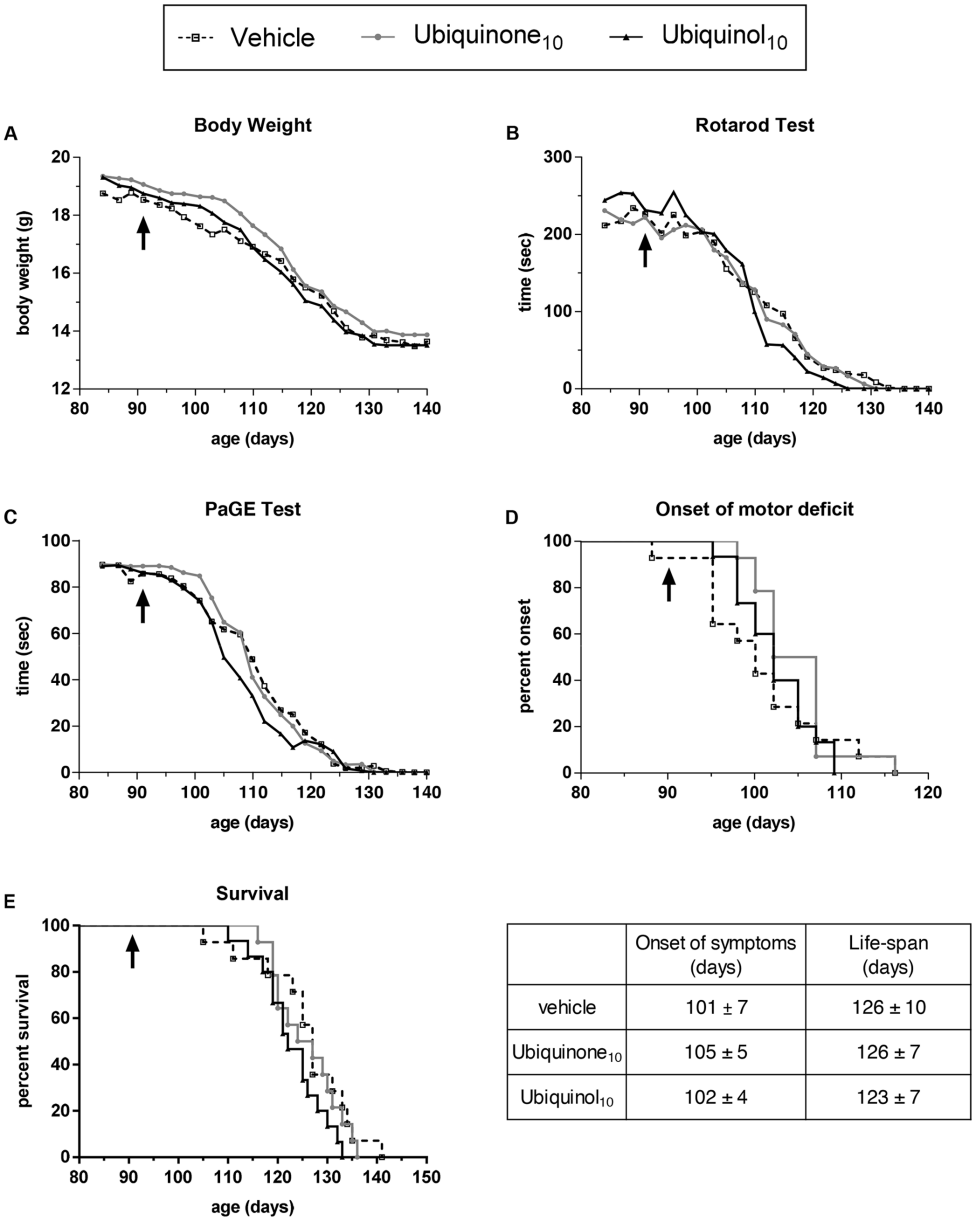
Ubiquinol_10_ chronic treatment has no effect on disease course in SOD1^G93A^ mice. Effect of oral treatment with 800 mg/kg/day ubiquinone_10_, ubiquinol_10_ or vehicle, on motor dysfunction and disease progression of 129Sv SOD1^G93A^ mice (n = 15 mice per group). Treatment started at the age of 91 days (arrows) until the sacrifice (at the end-stage of the disease, when mice were unable to right themselves within 10 seconds after being placed on both sides). Treatment with ubiquinone_10_ or ubiquinol_10_ had no significant effects on body weight (A), on the latency of rotarod (B) and PaGE test (C) (Two-way ANOVA), or on disease onset (D) and survival length (E). Each point represents the mean; for sake of clarity standard deviations are not indicated but they were always less than 15% of the value. Table reports the mean and standard deviations of symptoms onset and life-span for each group.

All mice were maintained at a temperature of 21±1°C with a relative humidity 55±10% and 12 h of light. Food (standard pellets) and water were supplied ad libitum.

Ubiquinone_10_ (Ubidecarenone from Kaneka) and ubiquinol_10_ (Kaneka QH™ from Kaneka) were administered by gavage after solubilization in sunflower seed oil (vehicle); preparation procedures were carried out at room temperature protecting from light. All the solutions were administered within four hours from preparation.

#### Ethics statement

Procedures involving animals and their care were conducted in conformity with the institutional guidelines of “Mario Negri” institute (IRFMN), that are in compliance with national (D.L. no. 116, G.U. suppl. 40, Feb. 18, 1992, Circular No.8, G.U., 14 luglio 1994) and international laws and policies (EEC Council Directive 86/609, OJ L 358, 1 Dec.12, 1987; NIH Guide for the Care and use of Laboratory Animals, U.S. National Research Council, 1996). All the experiments and the protocol proposed in the projects were reviewed and approved by the IRFMN Animal Care and Use Committee (IACUC), that includes members “ad hoc” for ethical issues. The mice were bred and maintained in a SPF environment. Animals with substantial motor impairment had food on the cage bottom and water bottles with long drinking spouts. The survival time was defined as the time when the animals were unable to right themselves within 10 s after being placed on either side. At this point, the animals were deeply anesthetized with Equithesin and then sacrificed by decapitation before proceeding to the dissection of tissues for biochemical analyses.

#### Preliminary acute treatment in 129Sv mice

Two preliminary pharmacokinetic studies were carried out in non-transgenic 129Sv female mice aging 9-11 weeks. For the time-course study, mice were treated orally with 200 mg/kg of ubiquinone_10_ or ubiquinol_10_ and killed at various times thereafter (30, 60, 180, 360 min and 24 hours after dosing, four mice/group). In a separate single-dose study, ubiquinone_10_ and ubiquinol_10_ were administered orally at various doses (200, 400, 800 and 1600 mg/kg) and the animals (4 mice/dose) were killed 3 hours after dosing. Four mice were treated with vehicle alone.

#### Chronic treatment in 129Sv SOD1G93A mice and analysis of their motor dysfunction and survival

Female 129Sv SOD1^G93A^ mice were treated by oral gavage with ubiquinone_10_ or ubiquinol_10_ (800mg/kg), or vehicle (n = 15 each group). The treatment started when the mice were 13 weeks old, which is the mean age that the growth of their body weight starts to differentiate from that of non-transgenic littermates and they show first alterations of hindlimbs abduction when raised by tail. This occurs just before the appearance of motor impairments (onset of motor deficit). Mice were treated daily until the end-stage of the disease, when they were sacrificed (see below).

Disease progression was assessed two times a week from the start of the experiment by an operator blinded to the treatment. The parameters included body weight, latency on rotating bar (rotarod) and paw grip endurance (PaGE) and were evaluated as previously described [17]. The motor symptoms onset was determined by the first impairment in grip strength or on rotarod performance for two consecutive time points. The mice were sacrificed when they were unable to right themselves within 10 seconds after being placed on both sides. This time point was considered as the end-stage of the disease and was used to estimate the survival time.

Statistical analysis of the data of body weight, grip strength and rotarod performance was carried out by Two-way ANOVA for repeated measures (time) and different groups (treatments), followed by *post-hoc* Bonferroni’s test to compare the effect of treatments in respect to vehicle at each time point. The analysis was applied at the time points when all animals for each group were still alive in order to maintain the number per group balanced. The age at onset and the survival length were statistically evaluated by the Log-rank test to compare probabilities.

### HPLC Analysis of CoQ_10_ and CoQ_9_ Levels

Animals were sacrificed by decapitation. Blood was collected from the heart in heparinized tubes. After centrifugation at 5000 g for 10 min, plasma was collected and stored at −80°C. CNS tissues were removed immediately, blotted with paper to remove surface blood, quickly frozen in dry ice and stored at −80°C.

All solvents and reagents used were HPLC grade (Carlo Erba, Milan, Italy). Ubiquinone_10_, ubiquinone_9_, FeCl_3_, 1,4-benzoquinone and sodium perchlorate were purchased from Sigma-Aldrich (St. Louis, MO, USA).

#### Plasma levels of total CoQ_10_ and CoQ_9_ in treated SOD1^G93A^ mice

The method described by Mosca *et al*. [18] was used. Fifty µL of plasma were pre-incubated for 10 min at room temperature with 15 µL of 1,4-benzoquinone (2 mg/mL in ethanol), in order to allow a complete oxidation of ubiquinol_9/10_. The samples were then added to 235 µL of 1-propanol, vortexed and then centrifuged at 10000 g for 2 minutes. Two hundred µL of supernatant were then injected into the HPLC system with UV detection at 275 nm (Waters 1525 Binary Pump; Waters 2487 Dual λ Absorbance Detector; Waters mod. 717-plus autosampler). Separation was carried out using a Supelcosil LC 18-DB column (15×0.46 cm, i.d. 5 µm, Supelco Inc., Bellefonte, PA, USA), with a Supelcosil LC 18-DB Supelguard Cartridge (2×0.4 cm, i.d. 5 µm, Supelco Inc., Bellefonte, PA, USA) by isocratic elution with ethanol/methanol (65∶35 v/v) at flow rate of 1 mL/min. The total run time was 19 min and the retention times for CoQ_9_ and CoQ_10_ were 7.1 and 8.9 min, respectively.

Total levels of CoQ_10_ and CoQ_9_ were quantified by reference to calibration curves with ubiquinone_10_ or ubiquinone_9_, run in parallel with each analytical session. Calibration curves were linear in the range 0.2–3.2 µg/ml for both coenzymes, with r^2^ values >0.99.

#### CNS levels of total CoQ_10_ and CoQ_9_ in treated SOD1^G93A^ mice

CoQs were extracted from CNS tissues and measured by HPLC with UV detection at 275 nm, based on the method described by Kitano *et al.* [14], with slight modifications.

CNS tissues were homogenized in ultrapure water (1 g in 20 mL), aliquots of 0.15 mL were added to 1 mL of acetonitrile and the samples were centrifuged at 2500 rpm for 5 min; the supernatants were collected and 1 ml aliquots were added to 0.1 mL of 2% FeCl_3_ to allow the oxidation of ubiquinols to ubiquinones. After 20 minutes incubation at room temperature the extraction procedure started by adding 2 ml methanol, 0.5 mL purified water and 5 ml hexane, followed by 15 minutes shaking, centrifugation (1200 g, 5 min) and collection of the organic phase. This procedure was repeated twice and the combined extracts were evaporated to dryness, the residue was dissolved in 150 µL of water/methanol (20∶80 v/v), transferred in Eppendorf tubes, centrifuged (2500 rpm, 30 sec) and 100 µl were injected into the chromatographic system (Waters 1525 Binary Pump; Waters 2487 Dual λ absorbance detector; Waters mod. 717-plus autosampler). Separation was carried out on a Kinetex 2.6 μ PFP 100 Å column (100×4.6 mm, Phenomenex Inc., Torrance, CA, USA), with a HPLC KrudKatcher 5 μ ultra column in-line filter (Phenomenex Inc., Torrance, CA, USA), using gradient elution with water/methanol (20∶80 v/v; solvent A) and acetonitrile/methanol (4∶96 v/v; solvent B). During HPLC analysis the solvent gradient was programmed to operate at a flow rate of 1 mL/min for 25 minutes at the following gradient conditions: step1 - from the initial condition of 100% solvent A to 100% solvent B over 16 min; step 2 - from 100% solvent B to 100% solvent A over 1 min. Retention times of CoQ_9_ and CoQ_10_ were 17.8 and 18.3 minutes, respectively.

Total levels of CoQ_10_ and CoQ_9_ were quantified by reference to calibration curves with ubiquinone_10_ or ubiquinone_9_, run in parallel with each analytical session. Calibration curves were linear in the range 4.0–16.0 µg/ml (CoQ_10_) and 10.0–60.0 µg/ml (CoQ_9_), with r^2^ values >0.99.

#### CNS levels of ubiquinol_9/10_ and ubiquinone_9/10_

The method described by Takada *et al*. [19] was used with slight modifications. Tissues were homogenized (1 g in 10 mL of ultrapure water) and divided in two aliquots, one to measure total CoQ_9/10_ levels (aliquot A) and the other to measure ubiquinone_9/10_ only (aliquot B). 100 µL of aliquot A were added to 10 µL of ultrapure water and 60 µL of 1,4-benzoquinone (2 mg/mL in ethanol) to allow a complete oxidation of ubiquinol. In parallel, 100 µL of aliquot B were added to 10 µL of 10% Na_2_EDTA (to protect ubiquinol from oxidation), and 60 µL of ethanol. After 10 min, both aliquots were added to 750 µL of ethanol/hexane (2∶5, v/v), shaken, and centrifuged at 4000g for 3 min (4°C). The supernatants were dried under nitrogen flow, reconstituted with 100 µL of ethanol and 50 µL were injected into a HPLC system (Waters 1525 Binary pump; Waters mod. 717-plus autosampler) coupled to a coulometric detector (ESA Couluchem 5100A with ESA analytical cell mod. 5011). The electrochemical analytical cell consisted of a series of two coulometric electrodes: the first one (−650mV) was for ubiquinone_9/10_ reduction and the second one (+400mV) was for detection of CoQ_9/10_ in the reduced form. The chromatographic column was a reverse phase Supelcosil LC-18 DB (15×0.46 cm, i.d. 3 µm; Supelco Inc., Bellefonte, PA, USA). The separation was carried out in isocratic operational mode with ethanol/methanol/HClO_4_ (700∶300:1, v/v/v) +7% sodium perchlorate at 0.8 mL/min for 13 minutes. Retention times were 5.3 min (ubiquinol_9_), 6.3 min (ubiquinone_9_), 7.1 min (ubiquinol_10_) and 8.8 min (ubiquinone_10_).

Total levels of CoQ_9/10_ (aliquot A), and ubiquinone_9/10_ (aliquot B) were quantified by reference to calibration curves with ubiquinone_9/10_, run in parallel with each analytical session. Calibration curves were linear in the range 5–50 µg/g tissue (ubiquinone_10_) and 20–100 µg/g tissue (ubiquinone_9_), with r^2^ values >0.99. Endogenous levels of ubiquinol_9/10_ were obtained as difference between total CoQ_9/10_ levels (determined in fully oxidized aliquot A) and ubiquinone_9/10_ levels (determined in aliquot B).

All the procedures were carried out rapidly, on ice, with protection from light, and in the presence of Na_2_EDTA, to limit the possibility that endogenous ubiquinol oxidizes to ubiquinone during the sample preparation. In these conditions, we verified that the storage of CNS homogenate at 4°C, for up to one hour, did not affect ubiquinol/total CoQ ratios (data not shown). We also note that the ubiquinol/total CoQ ratios detected in our study are very similar to published data [3], [19], [20]. Most importantly, we operated to ensure reliable comparisons between the different groups (SOD1^G93A^, SOD1^WT^ and non-transgenic), always including matched samples in the same analytical session, with random processing.

#### Plasma levels of ubiquinol_9_ and ubiquinone_9_

The method described by Takada *et al*. [21] was used with slight modifications. Plasma samples were divided in two aliquots, one to measure total CoQ_9_ levels (aliquot A) and the other to measure ubiquinone_9_ only (aliquot B). 50 µL of aliquot A were added to 50 µL of ultrapure water and 25 µL of 1,4-benzoquinone (2 mg/mL ethanol), to allow a complete oxidation of ubiquinol_9_. In parallel, 50 µL of aliquot B were added to 50 µL of 10% Na_2_EDTA (to protect ubiquinol from oxidation), and 25 µL of ethanol. After 10 min, both aliquots were added to 750 µL of ethanol/hexane (2∶5, v/v), shaken, and centrifuged at 4000 g for 3 min (4°C). The supernatant was dried under nitrogen flow, reconstituted with 200 µL of H_2_O/2-propanol (1∶5, v/v) and 150 µL were injected in a HPLC system as reported for CNS samples. The chromatographic separation was carried out in isocratic operational mode with ethanol/methanol/H_2_O/HClO_4_ (700∶300:10∶1, v/v/v/v) +7% sodium perchlorate at 1 mL/min for 15 minutes. Retention times for ubiquinol_9_ and ubiquinone_9_ were 6.2 and 8.6 minutes, respectively.

Total levels of CoQ_9_ (aliquot A), and ubiquinone_9_ (aliquot B) were quantified by reference to calibration curves with ubiquinone_9_, run in parallel with each analytical session. Calibration curve was linear in the range 0.05–1.20 µg/mL, with r^2^ values >0.99. Endogenous levels of ubiquinol_9_ were obtained as difference between total CoQ_9_ levels (determined in fully oxidized aliquot A) and ubiquinone_9_ levels (determined in aliquot B).

All the procedures were carried out rapidly, on ice, with protection from light, and in the presence of Na_2_EDTA. The ubiquinol/total CoQ ratios detected in our study are very similar to published data [3], [19], [20]. Most importantly, we operated to ensure reliable comparisons between the different groups (SOD1^G93A^, SOD1^WT^ and NTg), always including matched samples in the same analytical session, with random processing.

## Results

### Mice Treatment with Ubiquinone_10_ and Ubiquinol_10_

Since available data indicated that the stabilized formulation of ubiquinol_10_ has a better oral bioavailability than ubiquinone_10_ in different animal species [14], [15], preliminary studies were carried out to verify if this was true also in 129Sv mice. With this aim, plasmatic CoQ_10_ levels were measured in wild-type mice treated acutely with both ubiquinol_10_ and ubiquinone_10_. Fig. S2A shows that, after oral treatment with 200 mg/kg**,** the area under the plasma concentration-time curve over the dosing interval (AUC_0−24h_), was 1.5-fold higher for ubiquinol_10_ (14.2 µg**^.^**h/mL) than ubiquinone_10_ (9.8 µg**^.^**h/mL). Fig. S2B shows the plasma CoQ_10_ levels in mice treated with different doses of the two compounds, three hours before sacrifice, confirming higher CoQ_10_ levels in the plasma of ubiquinol_10_–treated mice than in the plasma of ubiquinone_10_-treated mice, except at the highest dose tested (1600 mg/kg). In particular, treatment with 800 mg/kg ubiquinol_10_ resulted in CoQ_10_ levels double than those found after treatment with the same dose of ubiquinone_10_ (1.5±0.2 and 0.8±0.4 µg/mL, respectively, p<0.05 Student’s t test).

Brain CoQ_10_ levels (13 µg/g tissue on average) were not affected by acute treatment with either ubiquinone_10_ or ubiquinol_10_, at any time-point or any concentration (data not shown).

### Chronic Treatment with Ubiquinone_10_ and Ubiquinol_10_ in SOD1^G93A^ Mice

#### Effect on motor dysfunction, body weight and survival of SOD1^G93A^ mice

Fig. 1 shows that chronic oral treatment of SOD1^G93A^ mice with 800 mg/kg/day ubiquinone_10_ or ubiquinol_10_, started at the onset of disease symptoms, did not modify the motor impairment progression assessed by rotarod performance (fig. 1B) and grip strength (fig. 1C), in respect to the vehicle-treated mice. The progressive loss of body weight (fig. 1A) was also unaffected by both treatments. The lack of effect was clearly evidenced by the overlapping Kaplan-Meyer curves and the similar mean for motor symptom onset (fig. 1D) and survival length (fig. 1E).

#### Total CoQ_10_ and CoQ_9_ plasma levels

Table 1 reports the plasma concentrations of total CoQ_10_ and CoQ_9_ after chronic administration of ubiquinone_10_ and ubiquinol_10_ in the same SOD1^G93A^ mice used for behavioral tests. The last dose of both formulations was administered two hours before sacrifice.

**Table 1:** Levels of CoQ_9/10_ in the plasma of SOD1^G93A^ mice treated chronically with ubiquinone_10_ or ubiquinol_10_.

Chronic treatment	Total CoQ_10_ (µg/mL)	Total CoQ_9_ (µg/mL)
Vehicle	<0.20 (5)	1.63±0.40 (5)
Ubiquinol_10_	3.40±0.22 (13)	1.25±0.12 (13)
Ubiquinone_10_	3.03±0.26 (9)	1.19±0.20 (9)

Female 129Sv SOD1^G93A^ mice were treated orally with 800 mg/kg of ubiquinol_10_ or ubiquinone_10_, or vehicle (sunflower seed oil), once a day, starting from age of 91 days until the last stage of the disease (age of 125 days, on average) when they were sacrificed, two hours after the last treatment. Values are means±SEM of (n) mice.

As expected, no detectable levels of CoQ_10_ were found in mice chronically treated with vehicle (<0.20 µg/mL), whereas levels of 3.0 and 3.4 µg/mL were measured in the mice treated with ubiquinone_10_ and ubiquinol_10_, respectively (difference not statistically significant). Plasma levels of CoQ_9_ were not significantly affected by treatment with either ubiquinone_10_ or ubiquinol_10_ (table 1).

#### Total CoQ_10_ and CoQ_9_ CNS tissues levels

**Figure 2:**
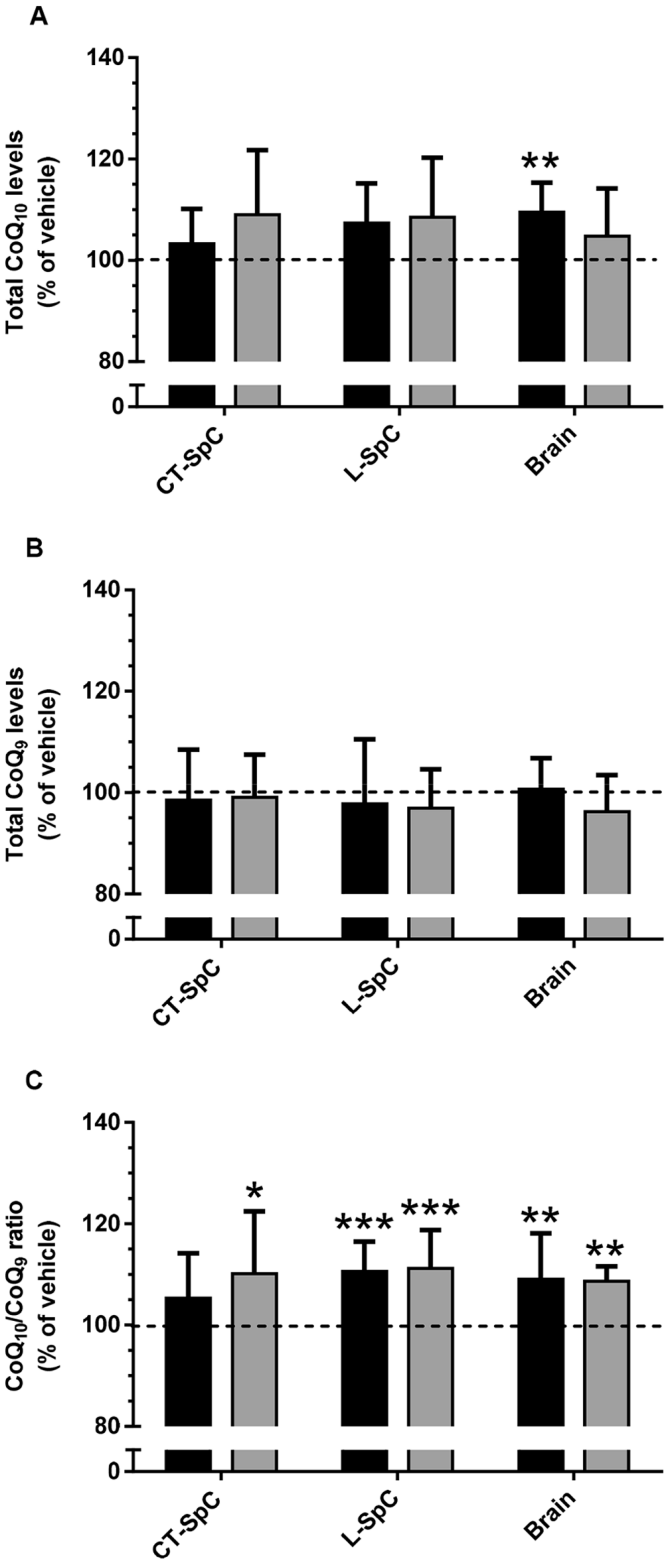
CoQ_9/10_ levels in brain and spinal cord of SOD1^G93A^ chronically treated with ubiquinol_10_ or ubiquinone_10_. Total levels of CoQ_10_ (A) and CoQ_9_ (B), and CoQ_10_/CoQ_9_ ratio (C), in brain, and in cervicothoracic (CT-SpC) and lumbar (L-SpC) spinal cord of mice chronically treated with 800 mg/kg ubiquinol_10_ (black) or ubiquinone_10_ (grey). Each value is the mean±SEM of 10–12 mice and is indicated as percentage of levels measured in mice treated with vehicle. * P<0.05, ** P<0.01 compared to vehicle (Dunnet’s multiple comparison tests following One-way ANOVA).

Figure 2 shows the levels of CoQ_10_ and CoQ_9_ measured in the brain and spinal cord (cervicothoracic, CT-SpC and lumbar, L-SpC) of SOD1^G93A^ mice after chronic treatment with ubiquinone_10_ or ubiquinol_10_, or vehicle.

CoQ_10_ levels in brain, CT-SpC and L-SpC of vehicle-treated mice were 17.6±0.3, 10.8±0.3 and 11.0±0.3 µg/g tissue (mean±SEM, n = 5), respectively; levels were slightly (4–11%) higher in all CNS tissues obtained from both ubiquinone_10_- or ubiquinol_10_-treated mice, with statistically significant differences detected in the brain of mice treated with ubiquinol_10_ only (Fig. 2A). CoQ_9_ levels were not affected by treatment with ubiquinone_10_ or ubiquinol_10_ (Fig. 2B). This finding prompted us to normalize, for each mouse, the CoQ_10_ level on the corresponding CoQ_9_ content, in order to minimize variability between mice. After this normalization, the levels of CoQ_10_ (expressed as CoQ_10_/CoQ_9_ ratio) appeared to be slightly but significantly increased (10%) by treatment with both ubiquinone_10_ or ubiquinol_10_ in all tissues, with the only exception for the levels in CT-SpC after treatment with ubiquinol_10_ (Fig. 2C).

### Levels of Reduced and Oxidized Forms of CoQ_10_ and CoQ_9_ in SOD1^G93A^ Mice

**Figure 3:**
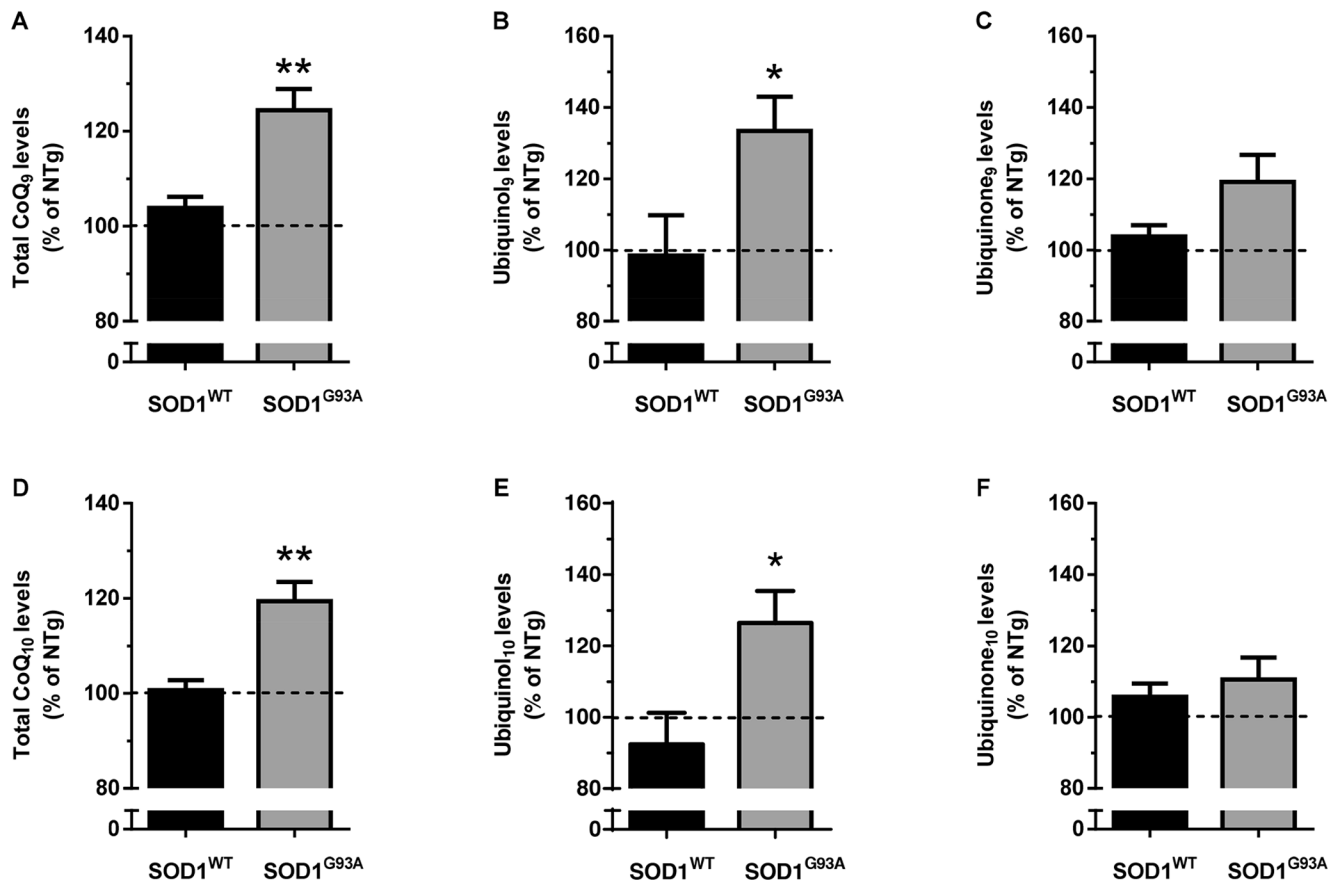
Increased levels of ubiquinol_9/10_ in the brain of symptomatic SOD1^G93A^ mice. Endogenous brain levels of CoQ_9_ (A–C) and CoQ_10_ (D–F) in 16 week-old male 129Sv SOD1^WT^ and SOD1^G93A^ mice are shown as percentage of the levels measured in age-matched 129Sv non-transgenic mice (absolute values in table S1). Panels A, D show the total CoQs levels, whereas panels B, E and panels C, F show the reduced and oxidized forms, respectively. Each value is the mean±SEM of 4–7 mice. * P<0.05, ** P<0.01 compared to SOD1^WT^ mice (Student’s T-test).

**Figure 4:**
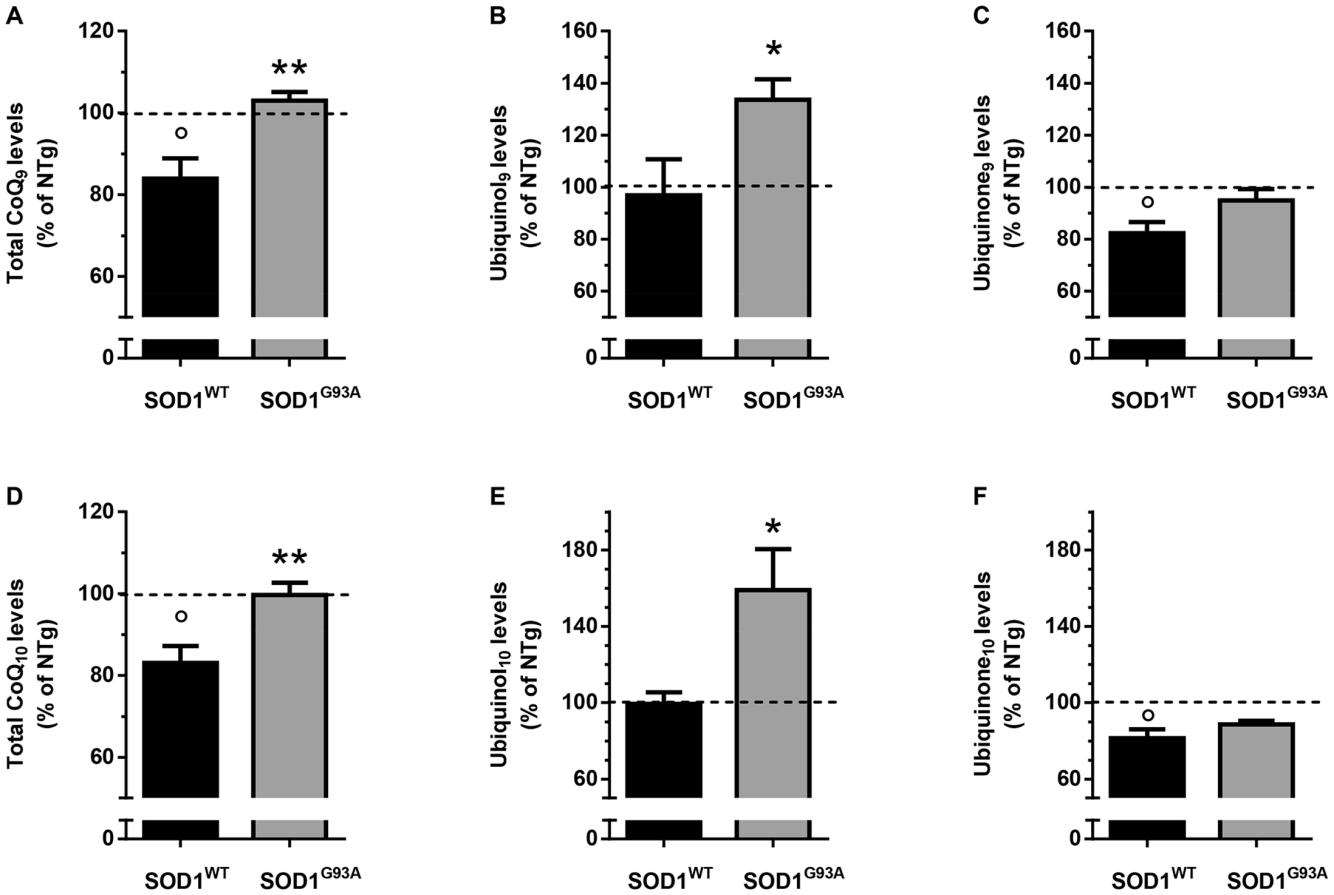
Increased levels of ubiquinol_9/10_ in the lumbar spinal cord of symptomatic SOD1^G93A^ mice. Endogenous levels of CoQ_9_ (A–C) and CoQ_10_ (D–F) in the lumbar spinal cord of male 16 week-old 129Sv SOD1^WT^ and SOD1^G93A^ mice are shown as percentage of the levels measured in age-matched 129Sv non-transgenic mice (absolute values in table S1). Panels A, D show the total CoQs levels, whereas panels B, E and panels C, F show the reduced and oxidized forms, respectively. Each value is mean±SEM of 4–7 mice. * P<0.05, ** P<0.01 compared to SOD1^WT^ mice; ° P<0.05 compared to 129Sv NTg mice (Student’s T-test).

**Figure 5:**
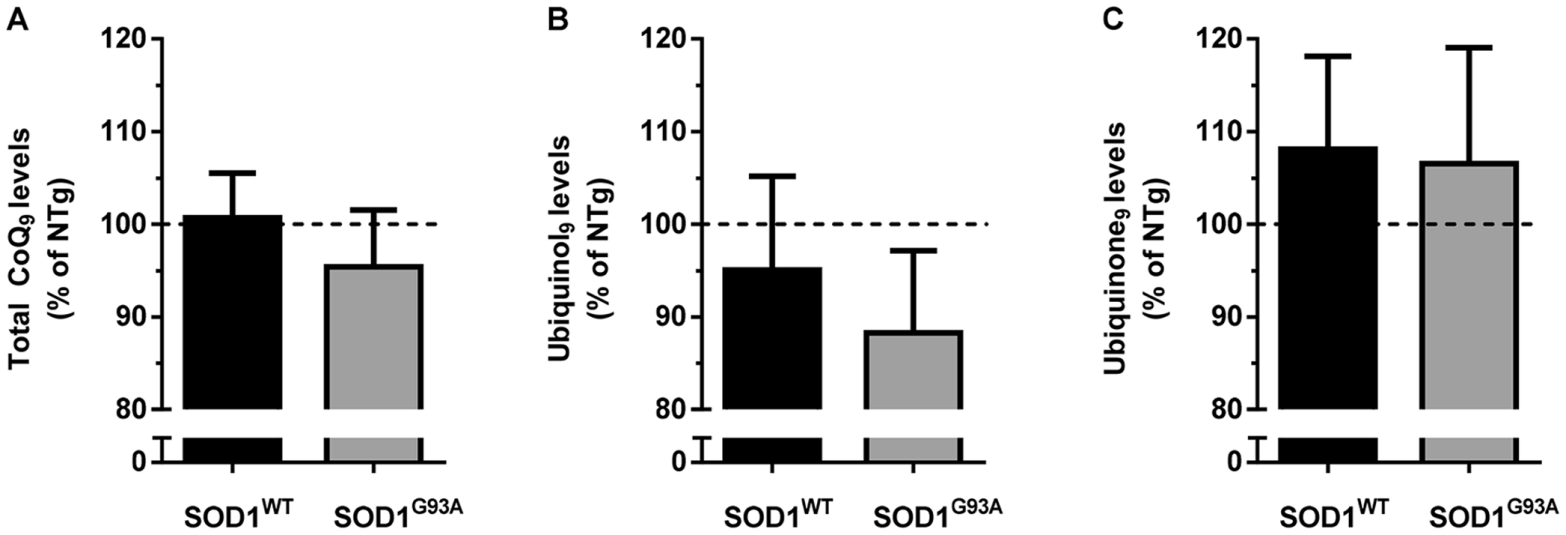
Unchanged plasma levels of ubiquinol_9_ in symptomatic SOD1^G93A^ mice. Levels of CoQ_9_ (A), ubiquinol_9_ (B) and ubiquinone_9_ (C) in plasma of male 16 week-old 129Sv SOD1^WT^ and SOD1^G93A^ mice are shown as percentage of the levels measured in age-matched 129Sv non-transgenic mice (table S1). Each value is mean±SEM of 4–7 mice.

Basal levels of reduced and oxidized forms of both coenzymes were measured in male 129Sv SOD1^G93A^ mice at the symptomatic stage of the disease (16 weeks), and in age-matched 129Sv SOD1^WT^ and non-transgenic 129Sv mice. In figures 3, 4, and 5 **,** the levels in transgenic mice are shown as a percentage of levels in non-transgenic mice (absolute values in table S1), in order to highlight the effect of the over-expression of the human SOD1 transgene, and any difference related to the G93A mutation.

#### Brain

Figure 3 shows that over-expression of human SOD1^WT^ enzyme does not alter the endogenous levels of oxidized and reduced CoQ_9/10_, in comparison with non-transgenic mice. Over-expression of the mutated protein (SOD1^G93A^) was instead associated with a significant increase (10–25%) of endogenous total levels of both CoQ_9_ and CoQ_10_, in comparison with age-matched SOD1^WT^ mice (Fig. 3A,D). This effect was associated with a significant increase (30–40%) of the reduced forms only (Fig. 3B,E), whereas no significant changes were observed for the oxidized forms (Fig. 3C,F).

#### Lumbar spinal cord

Endogenous levels of CoQ_9_ and CoQ_10_ in lumbar spinal cord of 16 weeks-old SOD1^WT^ mice were significantly lower (−20%), than in age-matched non-transgenic mice, a difference due to a decrease of the oxidized forms. In line with the results found in the brain, basal levels of CoQ_9_ and CoQ_10_ in SOD1^G93A^ were significantly higher (16–20%) than corresponding levels in age-matched SOD1^WT^ mice (Fig. 4A,D) and this increase was associated with a significant increase (36–60%) of the reduced forms only (Fig. 4B,E), whereas no or much lower changes where observed for the oxidized forms (Fig. 4C,F).

#### Plasma

Figure 5 shows that over-expression of human SOD1^WT^ enzyme does not alter the endogenous plasma levels of total CoQ_9_, ubiquinol_9_ and ubiquinone_9_, in comparison with non-transgenic mice. Moreover, no significant differences were observed between SOD1^WT^ and SOD1^G93A^ mice. CoQ_10_ levels were under the limit of detection.

## Discussion

Oxidative stress and mitochondrial impairment are the most prominent pathogenic mechanisms of ALS. The CoQ complex is a key component of the mitochondrial function, and its reduced form ubiquinol is fundamental for the antioxidant response, by direct interaction with radical species or by regenerating antioxidant agents [22].

It had been previously reported that chronic treatment with ubiquinone_10_ had a significant, although slight, effect on the life-span of a mouse model of familial ALS [12]. Regrettably, this finding was not confirmed in a phase II trial in humans [13]. One of the potential reasons underlying these results regarded the poor pharmacokinetic profile of ubiquinone_10_. In fact, a stabilized formulation of ubiquinol_10_, with a better bioavailability in respect to the oxidized form [14], [15] showed a better neuroprotective activity on MPTP-induced degeneration in mice [16].

On these basis we chronically treated a mouse model of familial ALS (SOD1^G93A^) with ubiquinol_10_, and ubiquinone_10_ in parallel, to increase CNS CoQ_10_ levels with the aim to obtain beneficial effect on the disease course.

Chronic treatment with both formulation resulted in a significant increase of plasmatic CoQ_10_ levels (from <0.2 to ≥3 µg/mL) that was associated to a very mild increase (10% on average) of the CoQ_10_ levels in CNS tissues (brain and spinal cord). Despite the better pharmacokinetic profile of the new formulation of ubiquinol_10_ after acute treatment [14,15 and present data], we found no differences in the CoQ_10_ levels after chronic treatment with both formulations.

No effect of the chronic treatments with either ubiquinone_10_or ubiquinol_10_ (both at 800 mg/kg/day) was observed on the disease progression or the life-span of SOD1^G93A^ mice.

This finding is at variance with previous data showing a 8% increase of survival after chronic treatment with a lower dose (200 mg/kg) of ubiquinone_10_ [12]. Unfortunately, that study did not report the levels of CoQ_10_ reached in the brain of SOD1^G93A^ mice after the chronic treatment. Two possible reasons may account for the discrepancy: 1) the strain of SOD1^G93A^ mice is different (C57BL6/SJL in [12] and 129Sv in our study), although the disease onset and progression is very similar in the two strains; and 2) in our study, the treatment started at the appearance of the first symptoms of disease (13 weeks of age), while Matthews *et al*. [12] started the treatment when mice were 7-weeks old, well before the onset of symptoms. According to the recent guidelines for preclinical animal research in ALS [23], the poor impact of preclinical studies on clinical practice is in part due to the fact that most of the pharmacological interventions are intended to be disease-preventive rather than being disease-modifying once the symptoms are evident. In this respect, since we start treatment when the mice exhibited the first disease symptoms, our data are in line with the lack of effect found in phase II clinical trials with ubiquinone_10_ [13].

The observation that the chronic treatments with ubiquinone_10_ and ubiquinol_10_ resulted in a very small increase of CoQ_10_ levels in the CNS of SOD1^G93A^ mice (≤10%) may provide an explanation for the lack of effect on disease progression and life span. In fact, it was previously shown that the neuroprotection observed after 1-week pretreatment of non-transgenic rats with 200 mg/kg ubiquinone_10_, i.e. prevention of the neurotoxicity induced by 3-nitropropionic acid, was associated with a ∼30% increase of brain CoQ_10_ [12]. All these data may suggest that an high enough increase of CNS concentrations of CoQ_10_ over basal levels is required to support neuroprotection and that the increase associated with our treatments in 129Sv SOD1^G93A^ mice, started at the time of the onset of symptoms, was too low to provide an efficient neuroprotection.

To better understand this point, in the second part of the study we measured the endogenous levels of CoQ_10_ and CoQ_9_ in CNS tissues and plasma of symptomatic SOD1^G93A^ mice, in comparison with age- and sex-matched SOD1^WT^ and non-transgenic mice.

Our results show for the first time that the CNS tissues of SOD1^G93A^ mice have CoQ_9/10_ levels 10–25% higher than those of age-matched SOD1^WT^ mice, while no differences were found in the plasma between the two strains. The increase was due to the reduced form only, ubiquinol, indicating a shift in the redox equilibrium toward a protective antioxidant state, and suggesting an attempt of the CNS to maintain an antioxidant environment to counteract the pathological processes of the disease. According with the antioxidant function of the reduced form of CoQ, it has been shown that ubiquinol_10_ is involved in the control of the mitochondrial levels of nitric oxide [24] and that the increase of ubiquinol pool in mitochondria (by antimycin) results in a decrease of protein nitration induced by ONOO^−^ [25]. Notably, increased protein tyrosine nitration has been observed in spinal cord of ALS transgenic mice (C57BL/6 SOD1^G93A^), from the pre-symptomatic till late stages of the disease, suggesting a role of nitrative stress in ALS pathogenesis and progression [26], [27]. Therefore, the increased levels of ubiquinol in the spinal cord of SOD1^G93A^ mice are likely not sufficient to counteract the intense nitrative stress occuring in these mice, with consequent lack of effect on their disease progression and life span.

An increase of ubiquinone_10_ with shift in the redox state of the coenzyme has been reported in the plasma [9] and CSF [10] of sALS patients and was suggested as a marker of the underlying oxidative stress. Our data, showing that the increase of ubiquinol_9/10_ in the CNS of SOD1^G93A^ mice was not paralleled by changes in plasma levels, indicate that the peripheral and central compartments react differently to the oxidative stress induced by mutant SOD1, and suggest that plasmatic CoQ levels do not represent sensitive markers for the changes associated to oxidative stress in the CNS. This is in line with the observations of Kontush *et al*. [28] who could not detect changes of ubiquinol_10_ in the plasma of patients with Alzheimer’s disease (AD), another neurodegenerative condition associated with increased free radical production in the CNS, even if higher levels of total CoQ were reported in post mortem brain tissues from AD patients [29].

In summary, our data show that chronic treatment of SOD1^G93A^ mice with ubiquinone_10_ or ubiquinol_10_ had no effect on the disease progression. This lack of efficacy may be due to the negligible changes of CoQ_9/10_ levels in the brain and spinal cord of treated mice, which in turn may be the consequence of a poor CNS availability of the compounds and/or the fact that endogenous CoQ_9/10_ levels are already significantly increased in SOD1^G93A^ mice.

## Supporting Information

Figure S1 **No gender effect on disease progression in SOD1^G93A^ mice.** Disease progression and survival length in males (gray) and females (black) SOD1^G93A^ mice on 129Sv background. Each point represent the mean of n = 10 male and n = 13 female. Table reports the mean and standard deviations of symptoms onset, life-span and disease duration for each group.
(TIF)

Figure S2 **Ubiquinol_10_ shows a better oral pharmacokinetic profile than ubiquinone_10_.** Plasma CoQ_10_ levels in female 129Sv non-transgenic mice treated orally with ubiquinone_10_ (grey) or ubiquinol_10_ (black). (A) Time-course after acute treatment with 200 mg/kg. (B) Dose-dependency after acute treatment with 200, 400, 800 and 1600 mg/kg and sacrifice 3 hours later. Each value is the mean±SEM of 4 mice. Plasma basal levels of CoQ_10_ were under the limit of quantification (<0.2 µg/mL).
(TIF)

Table S1 **Endogenous levels of CoQ_9/10_ in 129Sv NTg mice**. Endogenous levels (µg/g tissue or ng/mL) of total CoQ_9/10_, ubiquinol_9/10_ and ubiquinone_9/10_ in brain, lumbar spinal cord (L-SpC) and plasma of 16 week-old male 129Sv non-transgenic mice. Values are expressed as means±SEM of (n) mice.
(DOC)
