# Supplementary figures and images for: A Mouse Model of Familial ALS Has Increased CNS Levels of Endogenous Ubiquinol9/10 and Does Not Benefit from Exogenous Administration of Ubiquinol10
Source: PLoS One. 2013 Jul 23;8(7):e69540. doi: 10.1371/journal.pone.0069540 (PMC3720666; doi:10.1371/journal.pone.0069540)

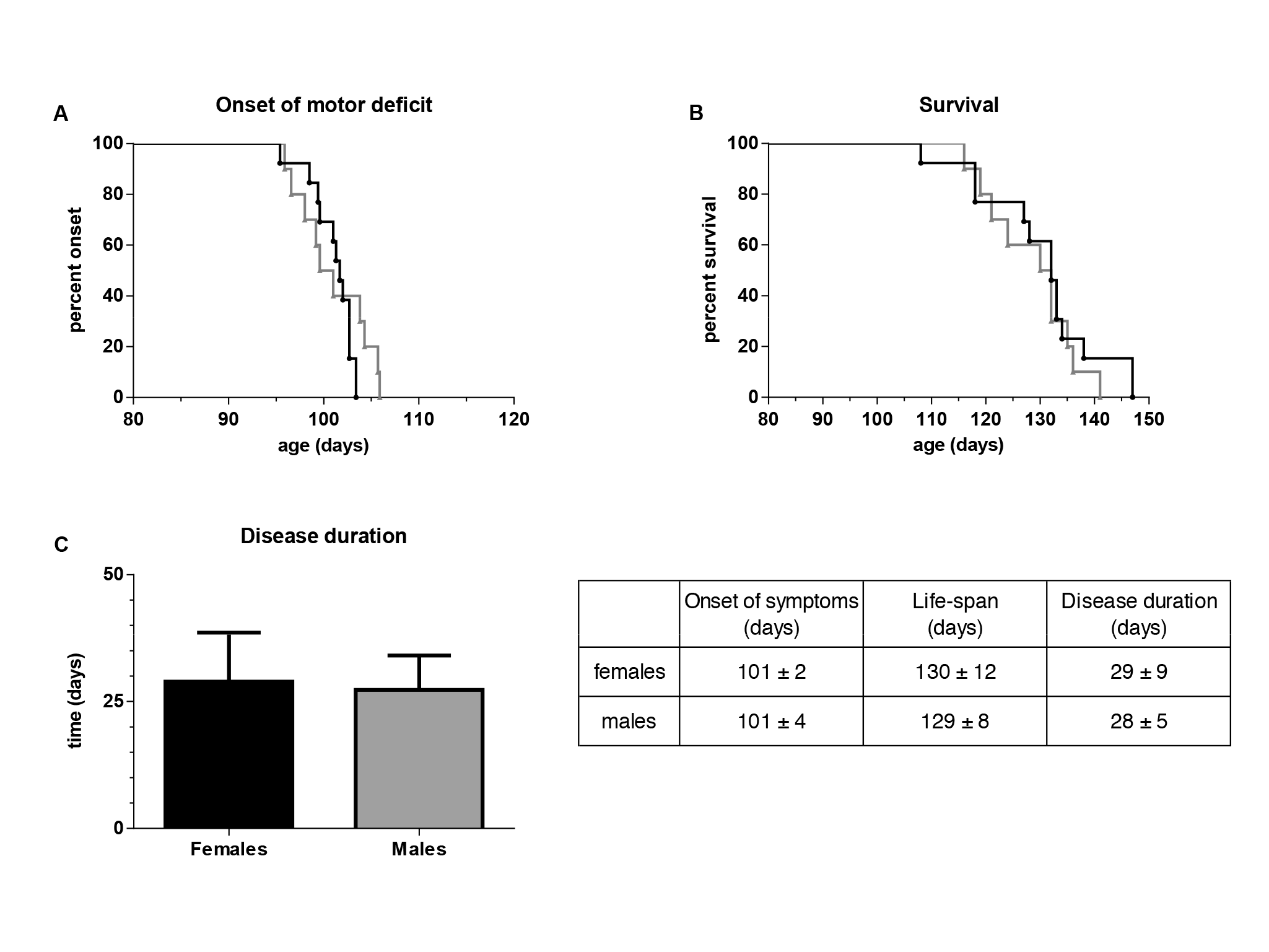

Supplement: Figure S1 — No gender effect on disease progression in SOD1G93A mice. Disease progression and survival length in males (gray) and females (black) SOD1G93A mice on 129Sv background. Each point represent the mean of n = 10 male and n = 13 female. Table reports the mean and standard deviations of symptoms onset, life-span and disease duration for each group. (TIF) [file pone.0069540.s001.tif]

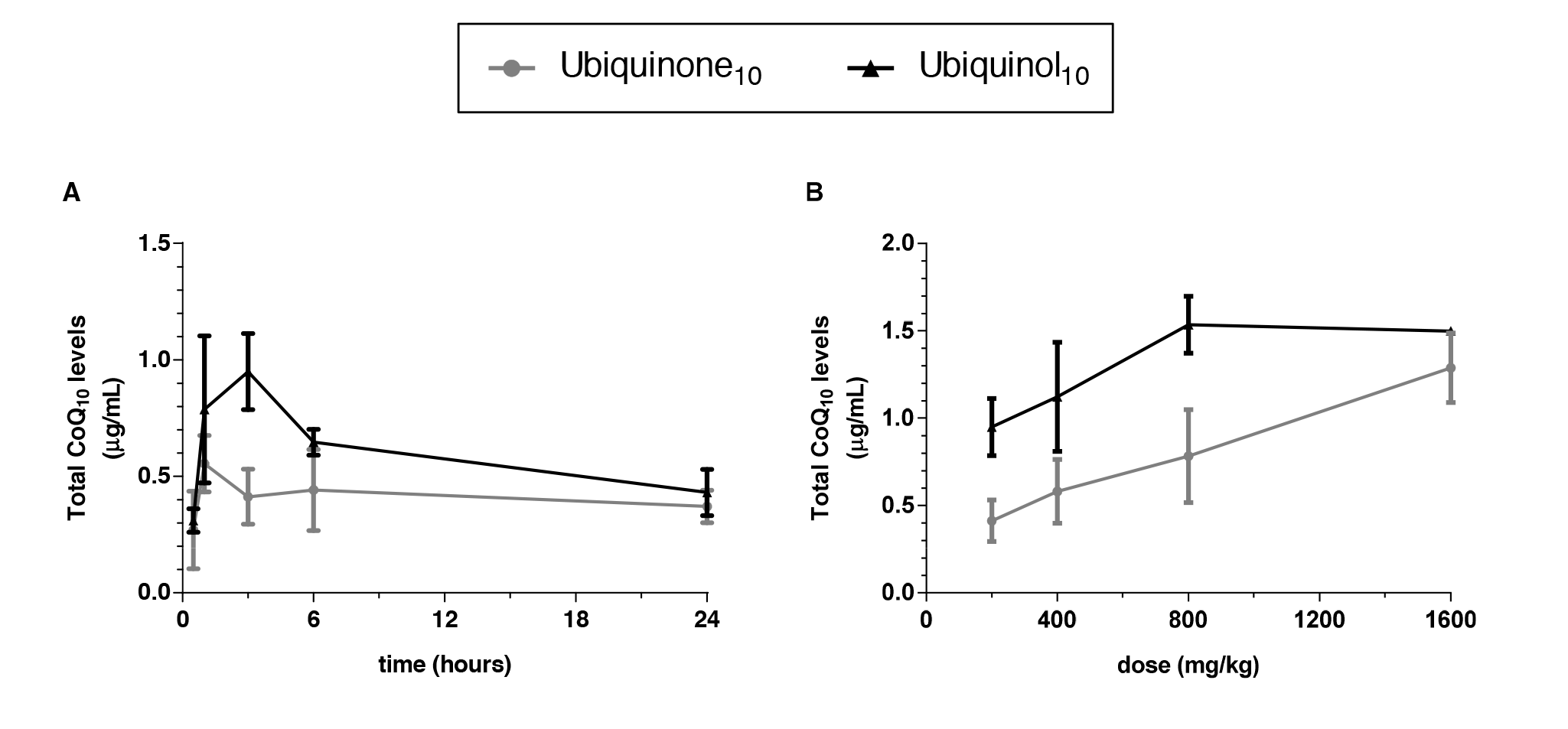

Supplement: Figure S2 — Ubiquinol10 shows a better oral pharmacokinetic profile than ubiquinone10. Plasma CoQ10 levels in female 129Sv non-transgenic mice treated orally with ubiquinone10 (grey) or ubiquinol10 (black). (A) Time-course after acute treatment with 200 mg/kg. (B) Dose-dependency after acute treatment with 200, 400, 800 and 1600 mg/kg and sacrifice 3 hours later. Each value is the mean±SEM of 4 mice. Plasma basal levels of CoQ10 were under the limit of quantification (<0.2 µg/mL). (TIF) [file pone.0069540.s002.tif]
