# Supplementary material for: A Mouse Model of Familial ALS Has Increased CNS Levels of Endogenous Ubiquinol9/10 and Does Not Benefit from Exogenous Administration of Ubiquinol10
Source: PLoS One. 2013 Jul 23;8(7):e69540. doi: 10.1371/journal.pone.0069540 (PMC3720666; doi:10.1371/journal.pone.0069540)
Supplement: Table S1 — Endogenous levels of CoQ9/10 in 129Sv NTg mice. Endogenous levels (µg/g tissue or ng/mL) of total CoQ9/10, ubiquinol9/10 and ubiquinone9/10 in brain, lumbar spinal cord (L-SpC) and plasma of 16 week-old male 129Sv non-transgenic mice. Values are expressed as means±SEM of (n) mice. (DOC) [file pone.0069540.s003.doc]

|  | **Total CoQ_9_** | **Ubiquinol_9_** | **Ubiquinone_9_** | **Total CoQ_10_** | **Ubiquinol_10_** | **Ubiquinone_10_** |
| --- | --- | --- | --- | --- | --- | --- |
| **Brain** | 41.6±1.6 (10) | 14.7±1.6 (10) | 26.9±1.5 (10) | 15.2±0.6 (10) | 5.7±0.6 (10) | 9.5±0.4 (10) |
| **L-SpC** | 34.5±1.4 (9) | 4.9±0.7 (9) | 29.6±0.8 (9) | 13.4±0.5 (8) | 1.8±0.2 (8) | 11.6±0.3 (8) |
| **Plasma** | 91.0±0.5 (5) | 56.0±4.0 (5) | 36.0±2.0 (5) | nd | nd | nd |
| nd: not detectable | | | | | | |
